# Analysis of the stochastic model for predicting the novel coronavirus disease

**DOI:** 10.1186/s13662-020-03025-w

**Published:** 2020-10-08

**Authors:** Ndolane Sene

**Affiliations:** Laboratoire Lmdan, Département de Mathématiques de la Décision, Faculté des Sciences Economiques et Gestion, Université Cheikh Anta Diop de Dakar, BP 5683 Dakar Fann, Senegal

**Keywords:** Stochastic numerical scheme, Novel coronavirus, Equilibrium points

## Abstract

In this paper, we propose a mathematical model to predict the novel coronavirus. Due to the rapid spread of the novel coronavirus disease in the world, we add to the deterministic model of the coronavirus the terms of the stochastic perturbations. In other words, we consider in this paper a stochastic model to predict the novel coronavirus. The equilibrium points of the deterministic model have been determined, and the reproduction number of our deterministic model has been implemented. The asymptotic behaviors of the solutions of the stochastic model around the equilibrium points have been studied. The numerical investigations and the graphical representations obtained with the novel stochastic model are made using the classical stochastic numerical scheme.

## Introduction

The spread of the novel coronavirus continues throughout the world. This novel coronavirus appeared in Wuhan and caused many inconveniences all over the world. Many people died in China, Italia, Espagna, Africa, and many countries worldwide due to this new disease. Presently, 20 June 2020, following the report 152 given by World Health Organization (WHO), the number of infected persons in the world is 8,525,042, and the number of deaths caused by this disease is 456,973 [1]. At this time, there exist no significant methods to stop this disease. One of the alternatives to prevent this disease is confinement and the use of masks, but these methods are not so efficient. This novel disease impacts many domains such as the economy, finance, and industries. There exist many models for predicting the novel coronavirus, but in our paper we use the SEIR model exposed in [2]. This novel coronavirus is known to behave very mysteriously and admits two types of symptoms: visible symptoms and invisible symptoms, making this disease very dangerous. Presently, the main problem with this disease is the infected persons who do not present symptoms. This category of infected persons continues to spread the disease and to infect other people throughout the world.

Nowadays, there exist many papers related to the prediction of the novel coronavirus. We give a brief review of the articles addressing this novel disease modeled with integer-order derivative and the non-integer-order derivative. In [3], Bozkurt et al. have introduced a new model for predicting the novel coronavirus. They mainly discussed a mathematical model of the evolution and spread of pathogenic coronaviruses from natural host to a human host. In [4], Monotosh et al. have introduced a model based on the dynamics of the novel coronavirus. In [5], Atangana has modeled the spread of COVID-19 with fractal-fractional operators and has asked the question: Can the lockdown save mankind before vaccination? In [6], Singh et al. have studied the novel coronavirus model by a new hybrid model of discrete wavelet decomposition and autoregressive integrated moving average models in application to one month forecasts of the casualties of COVID-19. In [7], Li et al. have proposed the SEIQDR model for predicting the novel coronavirus. In [8], Alkahtani et al. have studied the stability analysis and suggested a numerical scheme for their proposed model. There exist many investigations related to the epidemic models in general [9–11]. Many of the existing models in the literature can be adapted as well with the novel coronavirus with minors changes. For further references related to the novel coronavirus in fractional context, see [12–16]. There exist also many investigations related to the stochastic epidemic models; the reader may refer to [17–22].

In this paper, we try to model the novel coronavirus using differential equations. Due to the fact that this new disease is not controllable, we introduce in the new model the perturbations which take into account the other phenomena which are not detected with this new disease. In other words, we will focus on the stochastic novel coronavirus model. In this paper, we mainly investigate the positivity, the boundedness, and the stochastic stability around the equilibrium points of the novel coronavirus model. The primary tool used in this paper is the Ito lemma. The reproduction number is experienced because it is essential to control the novel coronavirus. By this number, we can give a proportion of the population infected by this novel virus to stop the disease. We also provide some illustrations of our main results by considering the data obtained in Senegal for a specific period. It is essential to mention that the stochastic model does not have equilibrium points in general. The method is to determine the equilibrium points of the deterministic model, and for the stochastic model, to study the asymptotic behaviors around the equilibrium points.

## Description of the model predicting the novel coronavirus

In this section, we propose a model which we use to study the novel coronavirus disease. We do not have many pieces of information related to the extinction of this disease in the world, but it is clear, based on the literature associated with this disease, and on the confirmed cases given by WHO related to the novel coronavirus, that we can use the SEIR model [2],

1 $$\begin{aligned}& \dot{S} = \Lambda -\mu S-nS (I+\psi A )-\eta SM, \end{aligned}$$

2 $$\begin{aligned}& \dot{E} = nS (I+\psi A )+\eta SM- \bigl( (1-\theta )w+\theta \rho +\mu \bigr)E, \end{aligned}$$

3 $$\begin{aligned}& \dot{I} = (1-\theta )wE- (\tau +\mu )I, \end{aligned}$$

4 $$\begin{aligned}& \dot{A} = \theta \rho E- (\gamma +\mu )A, \end{aligned}$$

5 $$\begin{aligned}& \dot{R} = \tau I+\gamma A-\mu R, \end{aligned}$$

6 $$\begin{aligned}& \dot{M} = \vartheta I+\varpi A-\pi M, \end{aligned}$$

with the initial conditions represented by the following relationships:

7 $$ S(0)=S_{0}, \qquad E(0)=E_{0}, \qquad I(0)=I_{0}, $$

and

8 $$ A(0)=A_{0}, \qquad R(0)=R_{0}, \qquad M(0)=M_{0}, $$

where the total population number is denoted by *N* at time *t* and is divided into six compartments: *S* denotes the susceptible, *E* represents the exposed people, *I* denotes the symptomatic infective, *A* represents the asymptotic infective, *R* indicates the recovered people and *M* represents the reservoir. Furthermore, the definitions of the parameters are summarized in the following list: Λ represents the birth–death rate of the people, *μ* represents the natural death rate of the people, *n* represents the disease transmission coefficient, *ψ* is the transmissibility multiplier of *A* to that *I*, *η* denotes the disease transmission coefficient from *M* to *S*, *θ* represents the proportion of asymptomatic infection, *ρ* and *w* indicate the transmission rate after completing the incubation period and becomes infected, joining the class *I* and *A*, the symptomatic people and the asymptomatic people joining the recovered class with the removal *τ* or recovery rate *γ*, respectively, *ϑ* and *ϖ* influence the symptomatic and the asymptomatic people contributing the virus into the seafood market *M*, and *π* is the removing rate of the virus from the seafood market. There are more pieces of information, the term $nSI$ designs the number of suspected persons infected by infective persons who enter in contact. The term *SA* designs the number of suspected persons infected by asymptomatic persons entering contacts. This term represents a problem in controlling the novel coronavirus. The issue is to test all the people massively when it is possible. But this issue is not always applicable due to the low status of some countries, where the capacity of hospitalization impacts is limited. An alternative to control this term in the world is confinement applied throughout the world. The time of incubation also influences this term; for example, asymptomatic people can be included in the recovery after incubation term when they have negative tests. The term *SM* is not applicable outside China because it stipulates the number of suspective persons that are infected when they enter in contact with the people in the market where the pandemic has begun. This term will be omitted in many countries like France, Senegal, Italy, USA, Switzerland, and Spain.

The trajectories generated by the model (1)–(8) can be experienced using numerical solutions; however, in many applications, the experimentally measured trajectories of the differential equations modeled by Eqs. (1)–(8) do not, in fact, behave as predicted. Therefore, to make our novel coronavirus model more realistic, we introduce the stochastic perturbation terms, including random noise types. The novel coronavirus is studied in the literature without perturbation terms. In this paper, we are interested in the novel coronavirus model when we introduce a white noise. In other words, how stochastic perturbations affect the deterministic model is described by Eqs. (1)–(8) in this paper. Due to some random environmental effects, for the rest of the paper, we consider the following perturbation terms: we replace in Eq. (1) *μ* by the term $\mu +\sigma _{1}\,dB_{1}$, in Eq. (2) *μ* by the term $\mu +\sigma _{2}\,dB_{2}$, in Eq. (3) *μ* by the term $\mu +\sigma _{3}\,dB_{3}$, in Eq. (4) *μ* by the term $\mu +\sigma _{4}\,dB_{4}$, in Eq. (5) *μ* by the term $\mu +\sigma _{5}\,dB_{5}$ and at last Eq. (6) *π* by the term $\pi +\sigma _{6}\,dB_{6}$. Note that the terms $B_{1}$, $B_{2}$, $B_{3}$, $B_{4}$, $B_{5}$, and $B_{6}$ denote independent Brownian motions and $\sigma ^{2}_{1}$, $\sigma ^{2}_{1}$, $\sigma ^{2}_{2}$, $\sigma ^{2}_{3}$, $\sigma ^{2}_{4}$, $\sigma ^{2}_{5}$ and $\sigma ^{2}_{5}$ represent the intensities of the associated stochastic perturbation terms. That is, we obtain the stochastic novel coronavirus model described by the following equations:

9 $$\begin{aligned}& d S = \bigl[\Lambda -\mu S-nS (I+\psi A )-\eta SM \bigr]\,dt+\sigma _{1}S\,dB_{1} (t ), \end{aligned}$$

10 $$\begin{aligned}& d E = \bigl[ nS (I+\psi A )+\eta SM- \bigl( (1- \theta )w+\theta \rho +\mu \bigr)E \bigr]\,dt+\sigma _{2}E\,dB_{2} (t ), \end{aligned}$$

11 $$\begin{aligned}& d I = \bigl[ (1-\theta )wE- (\tau +\mu )I \bigr]\,dt+\sigma _{3}I\,dB_{3} (t ), \end{aligned}$$

12 $$\begin{aligned}& d A = \bigl[\theta \rho E- (\gamma +\mu )A \bigr]\,dt+ \sigma _{4}A\,dB_{4} (t ), \end{aligned}$$

13 $$\begin{aligned}& d R = [\tau I+\gamma A-\mu R ]\,dt+\sigma _{5}S_{q}\,dB_{5} (t ), \end{aligned}$$

14 $$\begin{aligned}& d M = [\vartheta I+\varpi A-\pi M ]\,dt+\sigma _{6}R\,dB_{6} (t ). \end{aligned}$$

## Properties of the deterministic model for the novel coronavirus disease

In this section, we consider the deterministic model outside of China. In this context, the transmission coefficient *η* from *M* to *S* is considered null. Thus the novel coronavirus model under consideration is described by the following equation:

15 $$\begin{aligned}& \dot{S} = \Lambda -\mu S-nS (I+\psi A ), \end{aligned}$$

16 $$\begin{aligned}& \dot{E} = nS (I+\psi A )- \bigl( (1-\theta )w+ \theta \rho +\mu \bigr)E, \end{aligned}$$

17 $$\begin{aligned}& \dot{I} = (1-\theta )wE- (\tau +\mu )I, \end{aligned}$$

18 $$\begin{aligned}& \dot{A} = \theta \rho E- (\gamma +\mu )A, \end{aligned}$$

19 $$\begin{aligned}& \dot{R} = \tau I+\gamma A-\mu R, \end{aligned}$$

with the initial conditions represented by the following relationships:

20 $$ S(0)=S_{0}, \qquad E(0)=E_{0}, \qquad I(0)=I_{0}, \qquad A(0)=A_{0}, \qquad R(0)=R_{0}. $$

For the rest of the paper, we consider the $SEIA$ model because the equation for recovery of people *R* does not impact the other sub-equations.

### Positivity and boundedness of the solutions

In this section, we prove the positivity and the boundedness of the solutions using the standard method. We begin by the global positivity of the solutions. All the functions constituting the equations of the model are differentiable, continuous, and Lipschitz continuous, then the solutions of the model (15)–(20) exist and are unique. Our objective is to show the solutions are positive. We begin by the positivity of the variable *S*. First of all, we consider a time $t_{1}\geq 0$ verifying the set

21 $$ t_{1}=\min \bigl\{ t: S(t)=0, \text{ or } E(t)=0, \text{ or } I(t)=0, \text{ or } A(t)=0, \text{ or } R(t)=0 \bigr\} . $$

We first assume that $S(t_{1})=0$, which in turn implies that $S(t)<0$ for all $t\in [0,t_{1} ]$, then there exists a constant *A* such that

22 $$ \dot{S}\geq AS. $$

Thus there exists a positive function *n* such that

23 $$ \dot{S}(t)=AS(t)+n(t). $$

The solution of the above equation is given by the following equation:

24 $$ S(t)=S(0)\exp (At )+ \int ^{t}_{0}\exp \bigl(A(t-\tau ) \bigr)n(\tau )\,d \tau . $$

Neglecting the second positive term in Eq. (24), and considering the time $t_{1}$, we get the following relationship:

25 $$ S(t_{1})\geq S(0)\exp (At_{1} )>0, $$

which is in contradiction with $S(t_{1})=0$. That proves the solution *S* is positive for all $t \geq 0$. The same reasoning is repeated for the other variables of the model. Thus the solutions of the model (15)–(20) are positive. We finish by showing the boundedness of the solutions. Note that, by summing all the equations of the model (15)–(19), we have the following relationships:

26 $$ \frac{dN}{dt}=\Lambda -\mu N(t). $$

Applying the Laplace transform and its inverse to both sides of Eq. (26), we get the following solution:

27 $$ N(t)= \frac{\Lambda }{\mu } \bigl(1-\exp (-\mu t ) \bigr)+N(0) \exp (-\mu t ). $$

Thus the solutions are bounded; with the blow-up concept, we obtain the existence of the solutions for all $t\geq 0$. Furthermore, when *t* tends to +∞, then we have

28 $$ 0\leq N(t)\leq \frac{\Lambda }{\mu }. $$

### Reproduction number and its interpretations

In this section, we provide the reproduction number of the proposed model (15)–(20). We use the generation matrix method. Therefore we reduce the study by considering the variables *E*, *I*, and *A* because these variables are the infected variables. First of all we recall the trivial equilibrium point $(\frac{\Lambda }{\mu },0,0 )$ given by the relation below. We begin by recalling the matrix *F* and *V* at the trivial equilibrium point. They are represented in the following forms:

29 $$ F= \begin{pmatrix} 0&n\frac{\Lambda }{\mu }&n\psi \frac{\Lambda }{\mu } \\ 0&0& 0 \\ 0& 0 &0 \end{pmatrix} \quad \text{and}\quad V= \begin{pmatrix} \kappa &0&0 \\ - (1-\theta )w&\mu +\tau & 0 \\ -\theta \rho & 0 &\gamma +\mu \end{pmatrix}. $$

Then the reproduction number is obtained by determining the spectral radius of the matrix $FV^{-1}$. After calculation, we obtain the following relationship:

30 $$ \mathcal{R}_{0}= \frac{nc (1-\theta )w}{\kappa (\tau +\mu )}+ \frac{nc\psi \theta \rho }{\kappa (\gamma +\mu )}, $$

where $\kappa = (1-\theta )w+\theta \rho +\mu $ and $c=\frac{\Lambda }{\mu }$. The reproduction number plays an essential role in controlling the novel coronavirus disease; when this number is less than 1, we can consider the disease is controlled. But when this number exceeds 1, we can tell the disease is not controlled. This number also has another property; it can give us the maximal proportion of infected persons by the virus of corona under which the disease will die out. The following formula gives the percentage of the infected persons under which the novel coronavirus will die out:

31 $$ CIM\times 100= \biggl[1-\frac{1}{\mathcal{R}_{0}} \biggr]\times 100, $$

where $CIM$ denotes collective immunity, this option is not applied with the novel coronavirus due to the fact the percentage of death is not high, but the spread of this disease is exponential. Furthermore, the use of the mask also makes this option inapplicable in many countries because the masks and confinement can stop the spread of the disease considerably.

## Stochastic model for novel coronavirus disease

This section focuses on the spread of the novel coronavirus pandemic in the world. The advantage of using the stochastic model is motivated by the fact that this new disease is difficult to predict because we do not have an efficient therapy at this moment. Research on therapy continues around the world. The transmission by person to person is exponential. The children, in general, are rather not infected by this new virus. One in science tries to explain this fact in the literature. There exist many aspects that cannot be controlled with the novel coronavirus. Thus many perturbation terms can impact the mathematical modeling of the novel coronavirus. In this section, we study the stochastic novel coronavirus model with white noise. We will answer particularly to the boundedness of the solution in the first question. In the second question, we will focus on an extinction study and the asymptotic behavior around endemic equilibrium and the ergodicity. One of the main ideas is the use of the Ito lemma, which will be recalled in this paper. This section will be intense as regards calculations. The following equations describe the stochastic equation under consideration in this paper:

32 $$\begin{aligned}& d S = \bigl[\Lambda -\mu S-nS (I+\psi A ) \bigr]\,dt+ \sigma _{1}S\,dB_{1} (t ), \end{aligned}$$

33 $$\begin{aligned}& d E = \bigl[ nS (I+\psi A )- \bigl( (1-\theta )w+\theta \rho +\mu \bigr)E \bigr]\,dt+\sigma _{2}E\,dB_{2} (t ), \end{aligned}$$

34 $$\begin{aligned}& d I = \bigl[ (1-\theta )wE- (\tau +\mu )I \bigr]\,dt+\sigma _{3}I\,dB_{3} (t ), \end{aligned}$$

35 $$\begin{aligned}& d A = \bigl[\theta \rho E- (\gamma +\mu )A \bigr]\,dt+ \sigma _{4}A\,dB_{4} (t ), \end{aligned}$$

36 $$\begin{aligned}& d R = [\tau I+\gamma A-\mu R ]\,dt+\sigma _{5}S_{q}\,dB_{5} (t ). \end{aligned}$$

For preliminaries sets, we consider a probability space defined by $(\Omega ,F,P )$ with a filtration $\{ F_{t} \} _{t\geq 0}$ which is increasing and right continuous while $F_{0}$ contains all *P*-null sets. The Brownian motions defined by $B_{i}(t)$ where $i=1,2,3,4,5 $, are defined on the probability space. Let the space be defined by

$$ \mathbb{R}^{n}_{+}= \bigl\{ x\in \mathbb{R}^{n}: x_{i}>0, 1< i \leq n \bigr\} . $$

Let the stochastic differential equation be defined by the following equation:

37 $$ dx(t)=h (x,t )\,dt+g (x,t )\,dB(t), $$

with the initial condition represented by $x(0)=x_{0}\in \mathbb{R}^{n}$ and B(t) denotes the *n*-dimensional Brownian motion on the probability space $(\Omega ,F, \{ F_{t} \} _{t\geq 0},P )$ which is supposed complete. We define the operator *L* associated to Eq. (37) as follows:

38 $$ L=\frac{\partial }{\partial t}+\sum^{n}_{i=1}h_{i} (x,t ) \frac{\partial }{\partial x_{i}}+\frac{1}{2}\sum^{n}_{i,j=1} \bigl[g^{T} (x,t )g (x,t ) \bigr]_{ij} \biggl( \frac{\partial ^{2}}{\partial x_{i}\,\partial y_{i}} \biggr). $$

We define a fundamental operation for our investigations, *L* acts on the function $V\in C^{2,1} (\mathbb{R}^{n}\times [t_{0},\infty ], \mathbb{R}_{\geq 0} )$ by the following operation:

39 $$ LV(x,t)=\frac{\partial V}{\partial t}+V_{x} (x,t )h(x,t)+ \frac{1}{2} \operatorname{trace} \bigl[g^{T} (x,t )V_{xx} (x,t )g (x,t ) \bigr], $$

with

40 $$ V_{xx}= \biggl(\frac{\partial ^{2}V}{\partial x_{i}\,\partial x_{j}} \biggr)_{n\times n}, \qquad V_{x}= \biggl[ \frac{\partial V}{\partial x_{1}},\frac{\partial V}{\partial x_{2}},\ldots \biggr]. $$

The Ito lemma which will be used throughout stipulates the following relationship: if $x\in \mathbb{R}^{n}$, then we have

41 $$ dV(x,t)=LV (x,t )\,dt+V_{x} (x,t )g (x,t )\,dB (t ). $$

### Global positivity of the solutions of the novel coronavirus model

In this section, we will prove our model admits a unique positive solution which is bounded as well. For brevity, we establish the stochastic model related to novel coronavirus assuming it to be biologically well-posed. It is essential to mention that the sketch of the proofs for this section and the rest of the paper can be found in the literature, here we adapt the theorem with our novel model [17, 18, 20, 21].

#### Theorem 1

*Let the initial value* $(S (0 ),E (0 ),I (0 ),A (0 ),R (0 ) )^{T}\in \mathbb{R}_{\geq 0}$, *then the solution*

$$ \bigl(S (t ),E (t ),I (t ),A (t ),R (t ) \bigr)^{T}, $$

*for all strict positive time of the model* (32)*–*(36) *exists*, *and is positive almost surely*.

#### Proof

We assume that $(S (0 ),E (0 ),I (0 ),A (0 ),R (0 ) )^{T}\in \mathbb{R}_{\geq 0}$, then using the fact in our model all the functions are continuous, and Lipschitz continuous then there exists a positive solution $(S (t ),E (t ),I (t ),A (t ),R (t ) )$ into the interval $[0,\sigma _{e} ]$, where $\sigma _{e}$ denotes the explosion time. We have to prove the explosion time $\sigma _{e}=\infty $ almost surely. We consider natural number $k_{0}$ such that $S (0 )$, $E (0 )$, $I (0 )$, $A (0 )$, $R (0 )$ belongs to the interval $[\frac{1}{k_{0}},k_{0} ]$. For each $k\geq k_{0}$, where *k* is also a natural number, we define the stopping time in the following form:

42 $$ \begin{aligned}[b] \sigma _{k}&=\inf \biggl\{ \sigma \in [0,\sigma _{e} ]: \min \bigl\{ S(t),E(t),I(t),A(t),R(t) \bigr\} \leq \frac{1}{k} \text{ or }\\ &\quad {} \max \bigl\{ S(t),E(t),I(t),A(t),R(t) \bigr\} \geq k \biggr\} . \end{aligned} $$

We remark $\inf (\oslash )=\infty $. We notice the stopping time $\sigma _{k}$ defined in Eq. (42) increases as well when $k\rightarrow \infty $. Then two statements can be proven to hold almost surely. The first $\sigma _{\infty }=\lim_{k\rightarrow \infty }\leq \sigma _{e}$ almost surely and the second assumption is when $\sigma _{\infty }=\infty $ then $\sigma _{e}=\infty $ almost surely. For the rest of the proof, we suppose the statements are not satisfied, which in turn implies the existence of positive time *T* and $\zeta \in (0,1 )$ such that

43 $$ P (\sigma _{\infty }\leq T )\geq \zeta \quad \text{for all } k \geq k_{0}. $$

Let the function $V:\mathbb{R}^{3}\longrightarrow \mathbb{R}$ belong to the class $\mathcal{C}^{2}$. Let the function *V* be expressed in the following form:

$$\begin{aligned} V (S,E,I,A,R ) = & \biggl(S-c-c\ln \biggl(\frac{S}{c} \biggr) \biggr)+E-1-\ln (E )+I-1\\ &{}-\ln (I )+A-1- \ln (A )+R-1-\ln (R ), \end{aligned}$$

where the constant $c=\Delta /\mu $. We evaluate the stochastic derivative using the Ito lemma along the trajectories of Eqs. (32)–(36), and we have the following relationship:

44 $$\begin{aligned} dV = & \biggl(1-\frac{c}{S} \biggr)\,dS+ \biggl(1-\frac{1}{E} \biggr)\,dE+ \biggl(1-\frac{1}{I} \biggr)\,dI+ \biggl(1-\frac{1}{A} \biggr)\,dA+ \biggl(1- \frac{1}{R} \biggr)\,dR \\ &{}+ \frac{c\sigma ^{2}_{1}+\sigma ^{2}_{2}+\sigma ^{2}_{3}+\sigma ^{2}_{4}+\sigma ^{2}_{5}}{2}\,dt \\ =& \biggl(1-\frac{c}{S} \biggr) \bigl( \bigl[\Lambda -\mu S-nS (I+ \psi A ) \bigr]\,dt+\sigma _{1}S\,dB_{1} (t ) \bigr) \\ &{}+ \biggl(1-\frac{1}{E} \biggr) \bigl( \bigl[ nS (I+\psi A )- \bigl( (1-\theta )w+\theta \rho +\mu \bigr)E \bigr]\,dt+\sigma _{2}E\,dB_{2} (t ) \bigr) \\ &{}+ \biggl(1-\frac{1}{I} \biggr) \bigl( \bigl[ (1-\theta )wE- (\tau + \mu )I \bigr]\,dt+\sigma _{3}I\,dB_{3} (t ) \bigr) \\ &{}+ \biggl(1- \frac{1}{A} \biggr) \bigl( \bigl[\theta \rho E- (\gamma +\mu )A \bigr]\,dt+ \sigma _{4}A\,dB_{4} (t ) \bigr) \\ &{}+ \biggl(1-\frac{1}{R} \biggr) \bigl( [\tau I+\gamma A-\mu R ]\,dt+ \sigma _{5}S_{q}\,dB_{5} (t ) \bigr)+ \frac{c\sigma ^{2}_{1}+\sigma ^{2}_{2}+\sigma ^{2}_{3}+\sigma ^{2}_{4}+\sigma ^{2}_{5}}{2}\,dt \\ \leq & \biggl[\Delta +c\mu + (1-\theta )w+\theta \rho +4 \mu +\gamma +\tau + \frac{c\sigma ^{2}_{1}+\sigma ^{2}_{2}+\sigma ^{2}_{3}+\sigma ^{2}_{4}+\sigma ^{2}_{5}}{2} \biggr]\,dt \\ &{} +\sigma _{1} (S-c )\,dB_{1}(t)+\sigma _{2} (E-1 )\,dB_{2}(t)+\sigma _{3} (I-1 )\,dB_{3}(t) \\ &{}+\sigma _{4} (A-1 )\,dB_{4}(t)+\sigma _{5} (R-1 )\,dB_{5}(t). \end{aligned}$$

For simplification, we suppose the following constant: $K=\Delta +c\mu + (1-\theta )w+\theta \rho +4\mu +\gamma + \tau + \frac{c\sigma ^{2}_{1}+\sigma ^{2}_{2}+\sigma ^{2}_{3}+\sigma ^{2}_{4}+\sigma ^{2}_{5}}{2}$, then Eq. (44) can be re-expressed in the following form:

45 $$ \begin{aligned}[b] dV&\leq K\,dt +\sigma _{1} (S-c )\,dB_{1}(t)+\sigma _{2} (E-1 )\,dB_{2}(t)+\sigma _{3} (I-1 )\,dB_{3}(t)\\ &\quad {}+\sigma _{4} (A-1 )\,dB_{4}(t)+\sigma _{5} (R-1 )\,dB_{5}(t). \end{aligned} $$

We now integrate to both sides of Eq. (45) between 0 to $\sigma _{k}\wedge T$ and we calculate the expectation; we obtain the following inequality:

46 $$\begin{aligned} E \bigl[V \bigl(S (\sigma _{k}\wedge T ),E (\sigma _{k} \wedge T ),I (\sigma _{k}\wedge T ),A (\sigma _{k} \wedge T ),R (\sigma _{k}\wedge T ) \bigr) \bigr] \leq &V (0 )+E \int ^{\sigma _{k}\wedge T}_{0}K\,ds \\ \leq &V (0 )+KE (\sigma _{k}\wedge T ) \\ \leq &V (0 )+KT, \end{aligned}$$

where $V(0)=V (S (0 ),E (0 ),I (0 ),A (0 ),R (0 ) )$. We consider $\Omega _{k}= \{ \sigma _{k}\leq T \} $, for all $k\geq k_{1}$, which in turn implies from Eq. (43) that $P (\Omega _{k} )\geq \zeta $. We remark that, for every $w\in \Omega _{k}$, there exists at least

$$ S (\sigma _{k},w ), E (\sigma _{k},w ), I ( \sigma _{k},w ), A (\sigma _{k},w ), R (\sigma _{k},w ), $$

equaling the number *k* or $\frac{1}{k}$. We get

47 $$\begin{aligned} \begin{aligned}[b] &V \bigl(S (\sigma _{k}\wedge T,w ),E (\sigma _{k} \wedge T,w ),I (\sigma _{k}\wedge T,w ),A ( \sigma _{k}\wedge T,w ),R (\sigma _{k}\wedge T,w ) \bigr)\\ &\quad \geq \biggl( \frac{1}{k}-c-c\ln \biggl(\frac{c}{k} \biggr) \biggr)\wedge \bigl(k-1-\ln (k ) \bigr). \end{aligned} \end{aligned}$$

From Eq. (46) and Eq. (47), we obtain the following relationships:

48 $$\begin{aligned} \begin{aligned}[b] &V (0 )+KT\\ &\quad \geq E \bigl[1_{\Omega _{k}}V \bigl(S ( \sigma _{k}\wedge T,w ),E (\sigma _{k}\wedge T,w ),I (\sigma _{k}\wedge T,w ),A (\sigma _{k}\wedge T,w ),R (\sigma _{k}\wedge T,w ) \bigr) \bigr] \\ &\quad \geq \zeta \biggl(\frac{1}{k}-c-c\ln \biggl(\frac{c}{k} \biggr) \biggr)\wedge \bigl(k-1-\ln (k ) \bigr), \end{aligned} \end{aligned}$$

where $1_{\Omega _{k}}$ denotes the indicator function. We observe when $k\rightarrow \infty $

49 $$ \infty > V (0 )+KT=\infty , $$

which is absurd, which in turn implies $\sigma _{\infty }=\infty $, almost surely. That means the solution $(S, E, I, A, R)$ is globally positive, almost surely. □

### The remove of the novel coronavirus disease model

In this section, we study the asymptotic behavior around the trivial point given by $(\frac{\Delta }{\mu },0,0,0 )$. Concretely, we can observe that the trivial equilibrium is not an equilibrium point for the stochastic model (32)–(36). Thus, we cannot study the local stability of this point. The study made in this section gives us an alternative by studying the estimation of the average oscillation around this point because the model admits stochastic perturbation term. In this section, we focus on the extinction of the disease according to the value of the reproduction number. We present the following theorem to answer the above points.

#### Theorem 2

*Let the solution* $(S (t ),E (t ),I (t ),A (t ),R (t ) )^{T}$ *satisfy Eqs*. (32)*–*(36) *under the initial condition*

$$ \bigl(S (0 ),E (0 ),I (0 ),A (0 ),R (0 ) \bigr)^{T}\in \mathbb{R}_{\geq 0}, $$

*then when the reproduction number* $\mathcal{R}_{0}$ *is less than* 1, *the following relationship holds*:

50 $$ \lim_{t\rightarrow \infty }\sup \frac{1}{t} \int ^{t}_{0} \bigl(m_{1} \bigl(S(r)-c \bigr)^{2}+m_{2}E^{2}(r)+m_{3}I^{2}(r)+m_{4}A^{2}(r) \bigr)\,dr\leq \xi _{1}, $$

*where the constants are defined as follows*:

$$\begin{aligned}& m_{1} = 2\mu -\sigma ^{2}_{1}(1+c), \qquad m_{2}=\kappa - \frac{\sigma ^{2}_{2}}{2}, \qquad m_{3}=\tau +\mu - \frac{\sigma ^{2}_{3}}{2}, \\& m_{4}=\gamma +\mu - \frac{\sigma ^{2}_{4}}{2},\qquad \xi _{1}=\sigma ^{2}_{1}c(1+c), \end{aligned}$$

#### Proof

In this section, we utilize the change variables $x_{1}=S-c$, $x_{2}=E$, $x_{3}=I$, $x_{4}=A$. Then the stochastic model (32)–(36) can be rewritten in the following form:

51 $$\begin{aligned}& d x_{1} = \bigl[-\mu x_{1}-n (x_{1}+c )x_{3}-n\psi (x_{1}+c )x_{4} \bigr]\,dt+\sigma _{1} (x_{1}+c )\,dB_{1} (t ), \end{aligned}$$

52 $$\begin{aligned}& d x_{2} = \bigl[n (x_{1}+c )x_{2}+n\psi (x_{1}+c )x_{4}-\kappa x_{2} \bigr]\,dt+\sigma _{2}x_{2}\,dB_{2} (t ), \end{aligned}$$

53 $$\begin{aligned}& d x_{3} = \bigl[ (1-\theta )wx_{2}- (\tau +\mu )x_{3} \bigr]\,dt+\sigma _{3}x_{3}\,dB_{3} (t ), \end{aligned}$$

54 $$\begin{aligned}& d x_{4} = \bigl[\theta \rho x_{2}- (\gamma +\mu )x_{4} \bigr]\,dt+\sigma _{4}x_{4}\,dB_{4} (t ), \end{aligned}$$

where $\kappa = ( (1-\theta )w+\theta \rho +\mu )$. Let the function *V* be a class $\mathcal{C}^{2}$ function defined by the following:

55 $$ V (x )=V_{1} (x )+V_{2} (x )+V_{3} (x )+V_{4} (x ), $$

where the explicit form of the functions $V_{1}$, $V_{2}$, $V_{3}$, $V_{4}$ and $V_{5}$ are given by the following expressions:

56 $$ \begin{gathered} V_{1} (x )=\frac{x^{2}_{1}}{2}, \qquad V_{2} (x )= \frac{x^{2}_{3}}{2}, \qquad V_{3} (x )=\frac{x^{2}_{4}}{2}, \\ V_{4} (x )=x_{1}+x_{2}+ \frac{\kappa \mathcal{R}_{0}}{ (1-\theta )w} x_{3}, \qquad V_{5} (x )=\frac{ (x_{1}+x_{2} )^{2}}{2}. \end{gathered} $$

Our objective is to calculate $dV(x)=LV (x )\,dt+V_{x} (x )g (x )\,dB (t )$ represented in Eq. (41). We apply the Ito lemma along the trajectories of Eqs. (51)–(54), and we obtain the following expression:

57 $$\begin{aligned}& dV_{1}+dV_{2}+dV_{3} \\& \quad = LV_{1}\,dt+LV_{2}\,dt+LV_{3}\,dt+ \sigma _{1} (x_{1}+c )^{2}\,dB_{1}(t)+ \sigma _{3}x^{2}_{3}\,dB_{3}(t)+\sigma _{4}x^{2}_{4}\,dB_{4}(t), \end{aligned}$$

58 $$\begin{aligned}& dV_{4} = LV_{4}\,dt+\sigma _{1}x_{1}\,dB_{1}(t)+ \sigma _{2}x_{2}\,dB_{2}(t)+ \sigma _{3} \frac{\kappa \mathcal{R}_{0}}{ (1-\theta )w} x_{3}\,dB_{3}(t), \end{aligned}$$

59 $$\begin{aligned}& dV_{5} = LV_{5}\,dt+ (x_{1}+x_{2} ) \bigl[\sigma _{1} (x_{1}+c )\,dB_{1}(t)+\sigma _{2}x_{2}\,dB_{2}(t) \bigr]. \end{aligned}$$

For the rest of the proof, we will calculate $LV_{1}$, $LV_{2}$, $LV_{3}$, $LV_{4}$ and $LV_{5}$. The main idea in all the proofs is the use of the classical inequality $ab\leq a^{2}/2+b^{2}/2$. Following Eq. (39), we get the following results:

60 $$\begin{aligned}& \begin{aligned}[b] LV_{1}&=x_{1} \bigl[-\mu x_{1}-n (x_{1}+c )x_{3}-n\psi (x_{1}+c )x_{4} \bigr]+ \frac{\sigma ^{2}_{1} (x_{1}+c )^{2}}{2} \\ &=- \biggl(\mu -\frac{\sigma ^{2}_{1}}{2} \biggr)x^{2}_{1}+\sigma ^{2}_{1}cx_{1}+ \frac{\sigma ^{2}_{1}c^{2}}{2}-nx^{2}_{1}x_{3}-ncx_{1}x_{3}-n \psi x^{2}_{1}x_{4}-nc \psi x_{1}x_{4} \\ &\leq - \biggl(\mu -\frac{\sigma ^{2}_{1}(1+c)}{2} \biggr)x^{2}_{1}+ \frac{\sigma ^{2}_{1}c (1+c )}{2}-nx^{2}_{1}x_{3}-ncx_{1}x_{3}-n \psi x^{2}_{1}x_{4}-nc\psi x_{1}x_{4}, \end{aligned} \end{aligned}$$

61 $$\begin{aligned}& \begin{aligned}[b] LV_{2}&=x_{3} \bigl[ (1-\theta )wx_{2}- (\tau +\mu )x_{3} \bigr]+\frac{\sigma ^{2}_{3}x^{2}_{3}}{2} \\ &=- (\tau +\mu )x^{2}_{3}+ (1-\theta )wx_{2}x_{3}+ \frac{\sigma ^{2}_{3}x^{2}_{3}}{2} \\ &=- \biggl(\tau +\mu -\frac{\sigma ^{2}_{3}}{2} \biggr)x^{2}_{3}+ (1-\theta )wx_{2}x_{3}, \end{aligned} \end{aligned}$$

62 $$\begin{aligned}& \begin{aligned}[b] LV_{3}&=x_{4} \bigl[\theta \rho x_{2}- (\gamma +\mu )x_{4} \bigr]+\frac{\sigma ^{2}_{4}x^{2}_{4}}{2} \\ &=- (\gamma +\mu )x^{2}_{4}+\theta \rho x_{2}x_{4}+ \frac{\sigma ^{2}_{4}x^{2}_{4}}{2} \\ &=- \biggl(\gamma +\mu -\frac{\sigma ^{2}_{4}}{2} \biggr)x^{2}_{4}+ \theta \rho x_{2}x_{4}, \end{aligned} \end{aligned}$$

63 $$\begin{aligned}& \begin{aligned}[b] LV_{4}&=-\mu x_{1}-\kappa x_{2}+\kappa \mathcal{R}_{0}x_{2}- \frac{ (\tau +\mu )\kappa \mathcal{R}_{0}}{ (1-\theta )w} x_{3} \\ &=-\mu x_{1}+\kappa (\mathcal{R}_{0}-1 )x_{2}- \frac{ (\tau +\mu )\kappa \mathcal{R}_{0}}{ (1-\theta )w} x_{3}, \end{aligned} \end{aligned}$$

and finally

64 $$\begin{aligned} LV_{5} =& (x_{1}+x_{2} ) (-\mu x_{1}- \kappa x_{2} )+\frac{1}{2} \bigl[\sigma ^{2}_{1} (x_{1}+c )^{2}+ \sigma ^{2}_{2}x^{2}_{2} \bigr] \\ =&-\mu x^{2}_{1}-\kappa x_{1}x_{2}- \mu x_{1}x_{2}-\kappa x^{2}_{2}+ \frac{1}{2} \bigl[\sigma ^{2}_{1} (x_{1}+c )^{2}+\sigma ^{2}_{2}x^{2}_{2} \bigr] \\ \leq &- \biggl(\mu -\frac{\sigma ^{2}_{1}(1+c)}{2} \biggr)x^{2}_{1}- \biggl(\kappa -\frac{\sigma ^{2}_{2}}{2} \biggr)x^{2}_{2}+ \frac{\sigma ^{2}_{1}c (1+c )}{2}- (\kappa +\mu )x_{1}x_{2}. \end{aligned}$$

Under the assumption $\mathcal{R}_{0}\leq 1$ in Eq. (63) and summing Eqs. (60)–(64), we get the following inequality:

65 $$\begin{aligned} LV \leq &- \bigl(2\mu -\sigma ^{2}_{1}(1+c) \bigr)x^{2}_{1}- \biggl( \kappa -\frac{\sigma ^{2}_{2}}{2} \biggr)x^{2}_{2}- \biggl(\tau +\mu - \frac{\sigma ^{2}_{3}}{2} \biggr)x^{2}_{3}- \biggl(\gamma +\mu - \frac{\sigma ^{2}_{4}}{2} \biggr)x^{2}_{4}\\ &{}+\sigma ^{2}_{1}c(1+c). \end{aligned}$$

Thus, recalling the formula of $dV(x)=LV (x )\,dt+V_{x} (x )g (x )\,dB (t )$ represented in Eq. (41), it follows from the Ito lemma that

66 $$\begin{aligned} dV \leq & \bigl[-m_{1}x^{2}_{1}-m_{2}x^{2}_{2}-m_{3}x^{2}_{3}-m_{4}x^{2}_{4}+ \xi _{1} \bigr]\,dt \\ &{}+\sigma _{1} (x_{1}+c )^{2}\,dB_{1}(t)+ \sigma _{3}x^{2}_{3}\,dB_{3}(t)+ \sigma _{4}x^{2}_{4}\,dB_{4}(t) \\ &{}+\sigma _{1}x_{1}\,dB_{1}(t)+\sigma _{2}x_{2}\,dB_{2}(t)+\sigma _{3} \frac{\kappa \mathcal{R}_{0}}{ (1-\theta )w} x_{3}\,dB_{3}(t) \\ &{}+ (x_{1}+x_{2} ) \bigl[\sigma _{1} (x_{1}+c )\,dB_{1}(t)+ \sigma _{2}x_{2}\,dB_{2}(t) \bigr]. \end{aligned}$$

We integrate Eq. (66) between 0 to *t* and we apply at the same moment the expectation; we get the following relationship:

67 $$ EV \bigl(x(t) \bigr)-V \bigl(x(0) \bigr)\leq -E \int ^{t}_{0} \bigl[m_{1}x^{2}_{1}(r)+m_{2}x^{2}_{2}(r)+m_{3}x^{2}_{3}(r)+m_{4}x^{2}_{4}(r)- \xi _{1} \bigr]\,dr. $$

Using the fact the model (32)–(36) admits the positive bounded solution and using the changes variables, we arrive at the following inequality:

68 $$ \lim_{t\rightarrow \infty }\sup \frac{1}{t} \int ^{t}_{0} \bigl(m_{1} \bigl(S(r)-c \bigr)^{2}+m_{2}E^{2}(r)+m_{3}I^{2}(r)+m_{4}A^{2}(r) \bigr)\,dr\leq \xi _{1}. $$

The previous theorem can be interpreted thus: when $\mathcal{R}_{0}\leq 1$ in Eq. (63) and the parameters $m_{1}$, $m_{2}$, $m_{4}$, and $m_{4}$ are strictly positive, the solution of the stochastic model oscillates around the trivial equilibrium point, and the length of the oscillations are given by Eq. (50). □

### Asymptotic behavior around endemic equilibrium and ergodicity

In this section, we repeat the procedure of the previous section by considering the endemic equilibrium point. We mainly investigate the asymptotic behavior around the endemic equilibrium given by $(S^{\ast },E^{\ast },I^{\ast },A^{\ast } )$. We give the following theorem to answer the above point.

#### Theorem 3

*Let the solution* $(S (t ),E (t ),I (t ),A (t ),R (t ) )^{T}$ *satisfy Eqs*. (32)*–*(36) *under the initial condition*

$$ \bigl(S (0 ),E (0 ),I (0 ),A (0 ),R (0 ) \bigr)^{T}\in \mathbb{R}_{\geq 0}, $$

*then when the reproduction number* $\mathcal{R}_{0}$ *is greater than* 1, *the following relationship holds*:

69 $$ \begin{aligned}[b] &\lim_{t\rightarrow \infty }\sup \frac{1}{t} \int ^{t}_{0} \bigl(n_{1} \bigl(S(r)-S^{\ast } \bigr)^{2}+n_{2} \bigl(E(r)-E^{\ast } \bigr)^{2}+n_{3} \bigl(I(r)-I^{\ast } \bigr)^{2}+n_{4} \bigl(A(r)-A^{\ast } \bigr)^{2} \bigr)\,dr\\ &\quad \leq \xi _{2}, \end{aligned} $$

*where the constants are defined as follows*:

$$\begin{aligned}& n_{1} = 3\mu , \qquad n_{2}=3\kappa -\theta \rho - (1- \theta )w, \\& n_{3}= \frac{ (\tau +\mu ) (1-\theta )^{2}w^{2}-1}{ (1-\theta )^{2}w^{2}}, \qquad n_{4}= \frac{ (\mu +\gamma ) (\theta \rho )^{2}-1}{\theta ^{2}\rho ^{2}}, \end{aligned}$$

*and*

70 $$ \xi _{2}=\frac{2(\tau +\mu )}{ (1-\theta )^{2}w^{2}}I^{ \ast }+ \frac{(\tau +\mu )\sigma ^{2}_{4}I^{\ast }}{ (1-\theta )^{2}w^{2}}+ \frac{2(\gamma +\mu )}{ (\theta \rho )^{2}}A^{\ast }+ \frac{(\gamma +\mu )\sigma ^{2}_{4}A^{\ast }}{ (\theta \rho )^{2}}. $$

*In addition the solution of the stochastic novel coronavirus model is ergodic as well when the following inequality holds*:

71 $$ 0< \xi _{2}< \min \bigl\{ n_{1} \bigl(S^{\ast } \bigr)^{2},n_{2} \bigl(E^{ \ast } \bigr)^{2},n_{3} \bigl(I^{\ast } \bigr)^{2},n_{4} \bigl(A^{ \ast } \bigr)^{2} \bigr\} . $$

#### Proof

The first remark is to observe when the reproduction number is greater than 1; then the deterministic novel coronavirus disease model admits one endemic equilibrium point as described in the previous sections. We decompose our proof in five steps. In the first step, we consider the function $W_{1}$ to be of class $\mathcal{C}^{2}$ and defined by the following form:

72 $$ W_{1} (x )=\frac{1}{2} \bigl[S-S^{\ast }+E-E^{\ast }+A-A^{ \ast } \bigr]. $$

For simplification, we limit ourselves to the calculations of the function $LW_{1}$ according to the Ito lemma, and we have the following relationship:

73 $$\begin{aligned} LW_{1} =& \bigl[S-S^{\ast }+E-E^{\ast }+A-A^{\ast } \bigr] \bigl( \Delta -\mu S-\kappa E+\theta \rho E- (\mu +\gamma )A \bigr) \\ &{}+ \frac{\sigma ^{2}_{1}S^{2}}{2}+ \frac{\sigma ^{2}_{2}E^{2}}{2}+\frac{\sigma ^{2}_{4}A^{2}}{2} \\ =&-\mu \bigl(S-S^{\ast } \bigr)^{2}- (\kappa -\theta \rho ) \bigl(E-E^{\ast } \bigr)^{2}- (\mu +\gamma ) \bigl(A-A^{\ast } \bigr)^{2} \\ &{}- (\kappa -\theta \rho +\mu ) \bigl(S-S^{\ast } \bigr) \bigl(E-E^{\ast } \bigr)- (2\mu +\gamma ) \bigl(S-S^{ \ast } \bigr) \bigl(A-A^{\ast } \bigr) \\ &{}- (\mu +\gamma +\kappa -\theta \rho ) \bigl(A-A^{\ast } \bigr) \bigl(E-E^{\ast } \bigr)+\frac{\sigma ^{2}_{1}S^{2}}{2}+ \frac{\sigma ^{2}_{2}E^{2}}{2}+ \frac{\sigma ^{2}_{4}A^{2}}{2}. \end{aligned}$$

In the second step, we consider the function $W_{2}$ be a class $\mathcal{C}^{2}$ defined by the following form

74 $$ W_{2} (x )=\frac{1}{2} \bigl[S-S^{\ast }+E-E^{\ast }+I-I^{ \ast } \bigr]. $$

For simplification, we continue with the calculations of the function $LW_{2}$ according to the Ito lemma, and we have the following relationship:

75 $$\begin{aligned} LW_{2} =& \bigl[S-S^{\ast }+E-E^{\ast }+I-I^{\ast } \bigr] \bigl[ \Delta -\mu S-\kappa E+ (1-\theta )wE- (\tau +\mu )I \bigr] \\ &{}+ \frac{\sigma ^{2}_{1}S^{2}}{2}+ \frac{\sigma ^{2}_{2}E^{2}}{2}+\frac{\sigma ^{2}_{3}I^{2}}{2} \\ =&-\mu \bigl(S-S^{\ast } \bigr)^{2}- \bigl(\kappa - (1-\theta )w \bigr) \bigl(E-E^{\ast } \bigr)^{2}- (\tau +\mu ) \bigl(I-I^{\ast } \bigr)^{2} \\ &{}- \bigl(\kappa +\mu - (1-\theta )w \bigr) \bigl(S-S^{ \ast } \bigr) \bigl(E-E^{\ast } \bigr)- (\tau +2\mu ) \bigl(S-S^{\ast } \bigr) \bigl(I-I^{\ast } \bigr) \\ &{}- \bigl(\tau +\mu +\kappa - (1-\theta )w \bigr) \bigl(E-E^{ \ast } \bigr) \bigl(I-I^{\ast } \bigr)+\frac{\sigma ^{2}_{1}S^{2}}{2}+ \frac{\sigma ^{2}_{2}E^{2}}{2}+ \frac{\sigma ^{2}_{3}I^{2}}{2}. \end{aligned}$$

In the third step, we consider the function $W_{3}$ to be of class $\mathcal{C}^{2}$ and defined by the following form:

76 $$ W_{3} (x )=\frac{1}{2} \bigl[S-S^{\ast }+E-E^{\ast } \bigr]. $$

For simplification, we continue with the calculations of the function $LW_{3}$ according to the Ito lemma, and we have the following relationship:

77 $$\begin{aligned} LW_{3} =& \bigl[S-S^{\ast }+E-E^{\ast } \bigr] [\Delta - \mu S- \kappa E ]+\frac{\sigma ^{2}_{1}S^{2}}{2}+ \frac{\sigma ^{2}_{2}E^{2}}{2} \\ =&-\mu \bigl(S-S^{\ast } \bigr)^{2}-\kappa \bigl(E-E^{\ast } \bigr)^{2}- (\kappa +\mu ) \bigl(S-S^{\ast } \bigr) \bigl(E-E^{\ast } \bigr)+ \frac{\sigma ^{2}_{1}S^{2}}{2}+ \frac{\sigma ^{2}_{2}E^{2}}{2}. \end{aligned}$$

In the fourth step, we consider the function $W_{4}$ to be of class $\mathcal{C}^{2}$ and defined by the following form:

78 $$ W_{4} (x )=I-I^{\ast }-I^{\ast }\ln \biggl( \frac{I}{I^{\ast }} \biggr). $$

For simplification, we continue with the calculations of the function $LW_{4}$ according to the Ito lemma, and we have the following relationship:

79 $$\begin{aligned} LW_{4} =& \biggl(1-\frac{I^{\ast }}{I} \biggr) \bigl[ (1-\theta )wE- ( \tau +\mu )I \bigr]+ \frac{\sigma ^{2}_{4}I^{\ast }}{2} \\ =&\frac{ (1-\theta )wE}{I} \bigl(I-I^{\bullet } \bigr)- (\tau +\mu )I+ (\tau +\mu )I^{\ast }+ \frac{\sigma ^{2}_{4}I^{\ast }}{2}. \end{aligned}$$

Using the inequality $2ab\leq a^{2}+b^{2}$, the above equation can be written in the following form:

80 $$\begin{aligned}& LW_{4} \leq \frac{1}{2} \bigl(I-I^{\ast } \bigr)^{2}+ \frac{ (1-\theta )^{2}w^{2}E^{2}}{2I^{2}}- (\tau + \mu )I+ (\tau +\mu )I^{\ast }+ \frac{\sigma ^{2}_{4}I^{\ast }}{2}, \\& \begin{aligned}[b] \frac{2LW_{4}}{ (1-\theta )^{2}w^{2}} &\leq \frac{1}{ (1-\theta )^{2}w^{2}} \bigl(I-I^{\ast } \bigr)^{2}+\frac{E^{2}}{I^{2}}- \frac{2(\tau +\mu )}{ (1-\theta )^{2}w^{2}}I\\ &\quad {}+ \frac{2(\tau +\mu )}{ (1-\theta )^{2}w^{2}}I^{\ast }+ \frac{(\tau +\mu )\sigma ^{2}_{4}I^{\ast }}{ (1-\theta )^{2}w^{2}}. \end{aligned} \end{aligned}$$

In the last step, we consider the function $W_{5}$ to be of class $\mathcal{C}^{2}$ and defined by the following form:

81 $$ W_{5} (x )=A-A^{\ast }-A^{\ast }\ln \biggl( \frac{A}{A^{\ast }} \biggr). $$

For simplification, we continue with the calculations of the function $LW_{5}$ according to the Ito lemma, and we have the following relationship:

82 $$\begin{aligned} LW_{5} =& \biggl(1-\frac{A^{\ast }}{A} \biggr) \bigl[\theta \rho E- ( \gamma +\mu )A \bigr]+ \frac{\sigma ^{2}_{5}A^{\ast }}{2} \\ =&\frac{\theta \rho E}{A} \bigl(A-A^{\bullet } \bigr)- (\gamma + \mu )A+ (\gamma +\mu )A^{\ast }+ \frac{\sigma ^{2}_{5}A^{\ast }}{2}. \end{aligned}$$

Using the inequality $2ab\leq a^{2}+b^{2}$, the above equation can be written in the following form:

83 $$\begin{aligned}& LW_{5} \leq \frac{1}{2} \bigl(A-A^{\ast } \bigr)^{2}+ \frac{ (\theta \rho )^{2}E^{2}}{2A^{2}}- (\gamma + \mu )A+ (\gamma +\mu )A^{\ast }+ \frac{\sigma ^{2}_{5}A^{\ast }}{2}, \\& \frac{2LW_{5}}{ (\theta \rho )^{2}} \leq \frac{1}{ (\theta \rho )^{2}} \bigl(A-A^{\ast } \bigr)^{2}+\frac{E^{2}}{A^{2}}- \frac{2(\gamma +\mu )}{ (\theta \rho )^{2}}A+ \frac{2(\gamma +\mu )}{ (\theta \rho )^{2}}A^{\ast }+ \frac{(\gamma +\mu )\sigma ^{2}_{4}A^{\ast }}{ (\theta \rho )^{2}}. \end{aligned}$$

Let the function *W* be of class $\mathcal{C}^{2}$ and defined by the following form:

84 $$ W=W_{1}+W_{2}+W_{3}+\frac{2W_{4}}{ (1-\theta )^{2}w^{2}}+ \frac{2W_{5}}{ (\theta \rho )^{2}}. $$

We finish with the calculation of the function *LW* using the functions $LW_{1}$, $LW_{2}$, $LW_{3}$, $LW_{4}$ and $LW_{5}$, the upper bound of the function *LW* is given by the following inequality:

85 $$\begin{aligned} LW \leq &-n_{1} \bigl(S-S^{\bullet } \bigr)^{2}-n_{2} \bigl(E-E^{ \bullet } \bigr)^{2}-n_{3} \bigl(I-I^{\bullet } \bigr)^{2}-n_{4} \bigl(A-A^{\bullet } \bigr)^{2}+\xi _{2}, \end{aligned}$$

where

$$\begin{aligned}& n_{1} = 3\mu , \qquad n_{2}=3\kappa -\theta \rho - (1-\theta )w, \\& n_{3}= \frac{ (\tau +\mu ) (1-\theta )^{2}w^{2}-1}{ (1-\theta )^{2}w^{2}}, \qquad n_{4}= \frac{ (\mu +\gamma ) (\theta \rho )^{2}-1}{\theta ^{2}\rho ^{2}}, \end{aligned}$$

and

86 $$ \xi _{2}=\frac{2(\tau +\mu )}{ (1-\theta )^{2}w^{2}}I^{ \ast }+ \frac{(\tau +\mu )\sigma ^{2}_{4}I^{\ast }}{ (1-\theta )^{2}w^{2}}+ \frac{2(\gamma +\mu )}{ (\theta \rho )^{2}}A^{\ast }+ \frac{(\gamma +\mu )\sigma ^{2}_{4}A^{\ast }}{ (\theta \rho )^{2}}. $$

We integrate Eq. (85) between 0 to *t* and we apply at the same moment the expectation; we get the following relationship:

$$ \begin{aligned} &EW \bigl(x(t) \bigr)-W \bigl(x(0) \bigr)\\ &\quad \leq -E \int ^{t}_{0} \bigl[n_{1} \bigl(S(r)-S^{\ast } \bigr)^{2}+n_{2} \bigl(E(r)-E^{\ast } \bigr)^{2}+n_{3} \bigl(I(r)-I^{\ast } \bigr)^{2}+n_{4} \bigl(A(r)-A^{\ast } \bigr)^{2}- \xi _{2} \bigr]\,dr. \end{aligned} $$

From this and from the boundedness of the solution of the model (32)–(36), we conclude that Eq. (69) holds. We finish by proving the ergodicity of the solution of the model. Note that Eqs. (32)–(36) prove that the constants $n_{1}$, $n_{2}$, $n_{3}$, and $n_{4}$ are strictly positive, and we use the ellipsoid domain equation given by

87 $$\begin{aligned} n_{1} \bigl(S-S^{\bullet } \bigr)^{2}+n_{2} \bigl(E-E^{\bullet } \bigr)^{2}+n_{3} \bigl(I-I^{\bullet } \bigr)^{2}+n_{4} \bigl(A-A^{ \bullet } \bigr)^{2}\leq \xi _{2}, \end{aligned}$$

which lies in the domain $\mathbb{R}^{4}_{\geq 0}$. We consider any open neighborhood of the ellipsoid domain given by *U* such that $\bar{U}\subseteq \mathbb{R}^{4}_{\geq 0}$. Note that Eq. (85) implies $LW<0$ for all $x\in \mathbb{R}^{4}_{\geq 0}|U$. Furthermore the stochastic novel coronavirus disease model can be written its matrix form, that is,

$$ dx=f(x)\,dt+A(x)\,dB(t), $$

where the function *f* and the diffusion matrix are given by

88 $$ f(x)= \begin{pmatrix} \Lambda -\mu S-nS (I+\psi A ) \\ nS (I+\psi A )-\kappa E \\ (1-\theta )wE- (\tau +\mu )I \\ \theta \rho E- (\gamma +\mu )A \end{pmatrix}, \qquad A(x)= \begin{pmatrix} \sigma ^{2}_{1}S^{2}& 0&0&0 \\ 0&\sigma ^{2}_{2}E^{2}&0&0 \\ 0&0&\sigma ^{2}_{3}I^{2}&0 \\ 0&0&0&\sigma ^{2}_{4}A^{2} \end{pmatrix}. $$

There exists $M=\min \{ \sigma ^{2}_{1}S^{2},\sigma ^{2}_{2}E^{2},\sigma ^{2}_{3}I^{2}, \sigma ^{2}_{4}A^{2} \} $ such that the solution $(S,E,I,A )^{T}\in \bar{U}$ and $\epsilon = (\epsilon _{1},\epsilon _{2},\epsilon _{3},\epsilon _{4} )^{T}\in \mathbb{R}^{4}_{\geq 0}$ satisfying the relation

89 $$ \begin{aligned}[b] \sum^{4}_{i,j=1}&=\sigma ^{2}_{1}S^{2} \epsilon _{1}+\sigma ^{2}_{2}E^{2} \epsilon _{2}+\sigma ^{2}_{1}I^{2} \epsilon _{3}+\sigma ^{2}_{4}A^{2} \epsilon _{4}\\ &\geq \min \bigl\{ \sigma ^{2}_{1}S^{2}, \sigma ^{2}_{2}E^{2}, \sigma ^{2}_{3}I^{2}, \sigma ^{2}_{4}A^{2} \bigr\} \vert \epsilon \vert =M\epsilon ^{2}. \end{aligned} $$

From the previously established assumptions, we conclude that the stochastic novel coronavirus disease model admits the ergodic solution $(S,E,I,A )^{T}$. □

### Applications and illustrations

In this section, we give the application of the main results established in the previous sections. We represent the dynamics of the *E* the exposed population graphically, *I* the symptomatic infective and *A* asymptomatic infective in Senegal. We apply our study in the case of Senegal. We fix certain parameters:

• the natural mortality rate is equal to $\mu =0.0079\ \mathrm{day} ^{-1}$, the birth rate is considered proportional to the natural mortality rate, but here we estimate it to be $\Lambda =0.0079\ \mathrm{day} ^{-1}$;

• the contact rate or the disease transmission coefficient is assumed to be $n=0.4$;

• the transmission multiplier is estimated to be $\psi =0.8$, note that when this number converges to zero it means there is no transmission,

• the proportion of asymptomatic infection in our country is $\theta =0.6$, which tells how many persons are asymptomatic with the novel coronavirus disease;

• the incubation periods parameter is $w=\rho =1/10\ \mathrm{day} ^{-1}$, which means an infected person takes 10 days to present the first signs;

• the removal or recovery rate of *I*, $\tau =1/9\ \mathrm{day} ^{-1}$, that is, an infected person takes 9 days in general to recover when he receives specific treatment;

• and the removal or recovery rate of *A*, $\gamma =1/9\ \mathrm{day} ^{-1}$, which means an asymptomatic person takes 9 days in general to recover.

To analyze the extinction and the asymptotic behavior around the endemic point, we determine the generation number given by

90 $$ \mathcal{R}_{0}=2.9577>1. $$

We observe the reproduction number exceeds 1. It means, in general, that an infected person can contaminate three persons. We see when we prefer the collective immunity, the novel coronavirus will be stopped when the $CIM=66.2$. That explains the fact in Sénégal, the disease is continuing to cause deaths and infects persons. But the reproduction number $\mathcal{R}_{0}>1$, Theorem 3, means that the solutions of the stochastic model oscillate around the nontrivial equilibrium point with an amplitude $\xi _{2}$.

**Figure 1:**
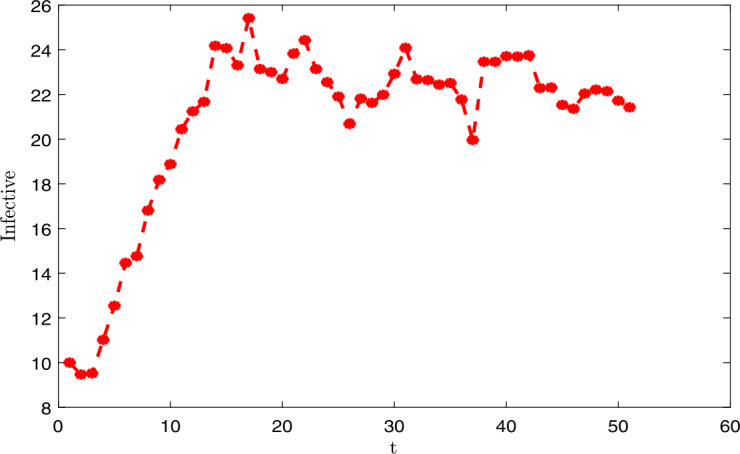
Dynamics of the infected individuals

**Figure 2:**
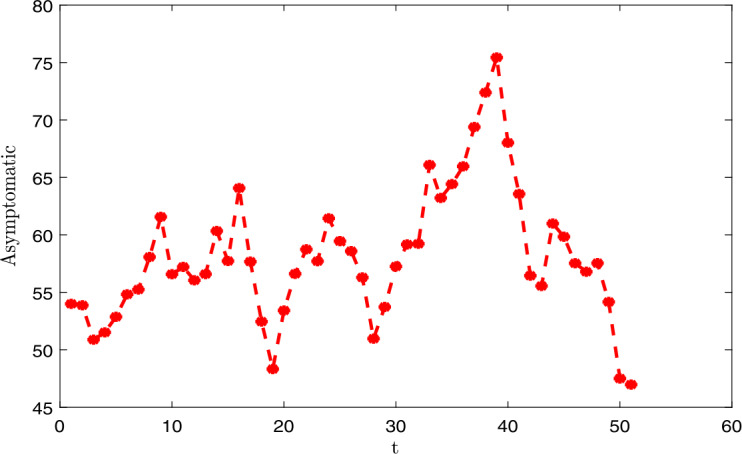
Dynamics of the asymptomatic individuals

**Figure 3:**
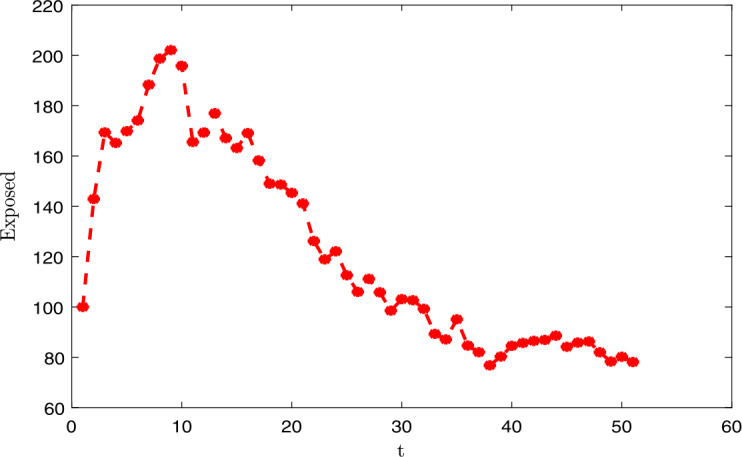
Dynamics of the exposed induviduals

We finish this section by proposing the numerical discretization and graphical representations. We use Monod–Milstein discretization, which is mostly used to discretize the stochastic differential. We have the following discretization:

91 $$\begin{aligned}& S_{k+1} = S_{k} \bigl[\Lambda -\mu S_{k}-nS_{k} (I_{k}+\psi A_{k} ) \bigr]\Delta t+\sigma _{1}S_{k}\chi _{1,k}\sqrt{\Delta t}+ \frac{\sigma ^{2}_{1}}{2}S_{k} \bigl(\chi ^{2}_{1,k}\Delta t-\Delta t \bigr), \end{aligned}$$

92 $$\begin{aligned}& E_{k+1} = E_{k}+ \bigl[ nS_{k} (I_{k}+ \psi A_{k} )- \kappa E_{k} \bigr]\Delta t+\sigma _{2}E_{k}\chi _{2,k}\sqrt{\Delta t}+ \frac{\sigma ^{2}_{2}}{2}E_{k} \bigl(\chi ^{2}_{2,k}\Delta t-\Delta t \bigr), \end{aligned}$$

93 $$\begin{aligned}& I_{k+1} = I_{k}+ \bigl[ (1-\theta )wE_{k}- (\tau + \mu )I_{k} \bigr]\Delta t+\sigma _{3}I_{k}\chi _{3,k}\sqrt{ \Delta t}+\frac{\sigma ^{2}_{3}}{2}I_{k} \bigl(\chi ^{2}_{3,k}\Delta t- \Delta t \bigr), \end{aligned}$$

94 $$\begin{aligned}& A_{k+1} = A_{k}+ \bigl[\theta \rho E_{k}- (\gamma +\mu )A_{k} \bigr]\Delta t+\sigma _{4}A_{k}\chi _{4,k}\sqrt{\Delta t}+ \frac{\sigma ^{2}_{4}}{2}A_{k} \bigl(\chi ^{2}_{4,k}\Delta t-\Delta t \bigr), \end{aligned}$$

95 $$\begin{aligned}& R_{k+1} = R_{k} [\tau I_{k}+\gamma A_{k}-\mu R_{k} ] \Delta t+\sigma _{5}R_{k} \chi _{5,k}\sqrt{\Delta t}+ \frac{\sigma ^{2}_{5}}{2}R_{k} \bigl(\chi ^{2}_{5,k}\Delta t-\Delta t \bigr), \end{aligned}$$

where $\chi _{1,k}$, $\chi _{2,k}$, $\chi _{2,k}$, $\chi _{2,k}$ and $\chi _{2,k}$ represent the independent Gaussian random variables with $N(0,1)$. We give the graphical representations (see in Figs. 1, 2, 3) with the following values of the intensities of the stochastic perturbations: $\chi _{1,k}=0.05$, $\chi _{2,k}=0.05$, $\chi _{2,k}=0.05$, $\chi _{2,k}=0.05$ and $\chi _{2,k}=0.05$. Note that all the values of the exposed, infective, asymptomatic cases depend on the intensity of the stochastic perturbations. In this section, we set $E=100$ (assumed), $I=10$ (comminatory transmission, they are infected because they present the novel coronavirus disease symptoms), $A=54$ (contact with infected persons). All the data of the disease are considered before June 1, 2020, in Senegal [1].

Note that to obtain the total estimated number of infected per day, we sum the number infective and the number of asymptomatic persons.
